# Characteristics of the microbiota in the nasopharynx and nasal cavity of healthy children before and during the COVID-19 pandemic

**DOI:** 10.1007/s12519-025-00953-z

**Published:** 2025-07-31

**Authors:** Min Liang, Wen-Jin Wu, Lei Li, Huan Qin, Shu-Na Li, Gui-Liang Zheng, Dong-Ming Hou, Qi Huang, Lan Cheng, Hui-Qun Jie, Jing-Rong Lu, Jing-Chun He, Jun Yang, Wei Wei

**Affiliations:** 1 Department of Otolaryngology-Head and Neck Surgery, Xinhua Hospital, Shanghai Jiao Tong University School of Medicine, Shanghai, China; 2 Ear Institute, Shanghai Jiao Tong University School of Medicine, Shanghai, China; 3 Shanghai Key Laboratory of Translational Medicine on Ear and Nose Disease, Shanghai, China

**Keywords:** Children, COVID-19 pandemic, Microbiota, Nasal cavity, Nasopharynx

## Abstract

**Background:**

Microbial colonization in the nasopharynx and nasal cavity plays a defensive role in children. The coronavirus disease 2019 (COVID-19) pandemic may have an influence on the nasopharynx and nasal cavity microbiota. This study aimed to identify and compare the microbiota in the nasopharynx and nasal cavity before and during the COVID-19 pandemic in a healthy pediatric population.

**Methods:**

Separate mucosal swabs were collected from the nasopharynx and nasal cavity of healthy children before and during the COVID-19 pandemic. A 16S ribosomal RNA-based metagenomic approach was employed to characterize and analyze alterations in the nasopharyngeal and nasal microbiota to determine whether isolation measures, such as mask wearing, influence microbial ecology.

**Results:**

The richness and diversity of the nasopharyngeal and nasal microbiota decreased during the COVID-19 pandemic compared with before the pandemic. *Firmicutes* and *Proteobacteria* were the most abundant phyla in the nasopharyngeal and nasal microbiota, respectively, both before and during the pandemic. *Corynebacterium* and *Moraxella* were the dominant genera in the nasopharyngeal and nasal microbiota during the COVID-19 pandemic, whereas *Pseudomonas* and *Corynebacterium* were dominant before the pandemic. Compared with pre-pandemic conditions, microbial colonization differed significantly for *Cyanobacteria/Chloroplast* and *Bacteroidetes* in the nasopharynx and for *Planctomycetes* in the nasal cavity during the COVID-19 pandemic.

**Conclusions:**

This study revealed a lower microbiota diversity during COVID-19, possibly accompanied by microbiota dysbiosis, increased risk of respiratory infections and inflammatory responses in healthy children. This study underscores the importance of reestablishing microbiota balance and highlights the need for personalized treatment and prophylactic strategies in routine public health practice.

Supplementary file3 (MP4 150533 KB)

**Supplementary Information:**

The online version contains supplementary material available at 10.1007/s12519-025-00953-z.

## Introduction

The nasopharyngeal and nasal microbiota play pivotal roles in the local immune defense against pathogenic bacteria and are closely related to human health and disease [1]. Microbiota dysbiosis at these sites is reportedly involved in many diseases in the pediatric population [2–4]. The nasopharyngeal and nasal microbiota are diverse, and the presence of opportunistic pathogens in the nasopharynx and nasal cavity is associated with adenoidal hypertrophy, tonsil hypertrophy, chronic rhinosinusitis, allergic rhinitis, otitis media, and severe obstructive sleep apnea (OSA) [5–12]. Many studies have focused on microbial colonization and analysis under pathological conditions in children. However, limited data exist from analyses of the nasopharyngeal and nasal microbiota in healthy individuals.

On January 30, 2020, the World Health Organization (WHO) declared coronavirus disease 2019 (COVID-19) a global health emergency [13]. During the pandemic, residents around the world, both adults and children, were told to wear masks for many hours daily to defend themselves against viruses. The WHO declared that COVID-19 was no longer a public health emergency of international concern on May 5, 2023 [14]. The use of masks may help reduce the spread of respiratory viruses and lower the probability of many respiratory infections [15–18]. However, very little is known about whether microbial colonization in the nasopharynx and nasal cavity has changed in healthy children before and during the COVID-19 pandemic.

The human nasopharyngeal and nasal cavities host complex bacterial communities that are mainly stable at the genus level but can vary between individuals. The bacterial communities in the nasopharynx and nasal cavity of children appear to differ from those in adults. Cultivation studies have revealed that potentially pathogenic bacteria such as *Streptococcus*, *Haemophilus influenzae*, *Neisseria*, *Staphylococcus aureus*, *Actinomyces*, *Porphyromonas*, *Bacteroides*, *Prevotella*, *Peptostreptococci*, and *Fusobacterium* are often isolated from the nasopharynx of healthy children [19]. Culture-independent molecular surveys based on 16S ribosomal RNA (rRNA) sequencing are currently being employed to investigate the microbiota on the skin surface and within the nasal cavity and nasopharynx [20–22]. The extracted DNA of sufficient quantity and quality was subjected to polymerase chain reaction (PCR) amplification of the bacterial 16S rRNA genes. These genes occur in all bacteria, and their analysis by sequencing or related approaches is a cornerstone of contemporary studies of microbial ecology [23]. This sequencing approach enables us to identify both abundant and rare members of the microbiome [24], rapidly changing our understanding of the microbiome in healthy and unhealthy children.

This study aimed to identify and compare the basic structure of the microbiota in the nasopharynx and nasal cavity before and during the COVID-19 pandemic in a healthy pediatric population via 16S rRNA pyrosequencing.

## Methods

### Study design and inclusion criteria

This retrospective study was carried out from February 2019 to August 2021. Children aged between four and eight years who were not diagnosed with adenoid hypertrophy were recruited for the study at the Department of Otolaryngology-Head and Neck Surgery, Xinhua Hospital, Shanghai Jiao Tong University School of Medicine, Shanghai, China. These children usually visited our hospital for transnasal electronic laryngoscopy for investigation of vocal nodules, and no adenoid hypertrophy was observed. The exclusion criteria for the children were as follows: (1) had a history of adenoidal hypertrophy; (2) presented with current otitis media, allergic rhinitis, acute/chronic nasosinusitis, or acute respiratory tract infections; (3) had a history of systemic or local glucocorticoids, antibiotics, or saline nasal irrigation within two months; and (4) had a history of tonsillectomy, adenoidectomy, or ventilation tube insertion. Five children who did not wear masks before the onset of COVID-19 were recruited from February 2019 to July 2019, and the other five, who wore masks during the pandemic, were recruited from July 2021 to August 2021. The proportion of children eligible for inclusion was 22.7% (5/22) in 2019 before the pandemic and 33% (5/15) in 2021 during the pandemic.

### Sample collection

All nasopharyngeal and nasal swab samples were collected via electronic nasopharyngoscopes after topical anesthesia. When a swab touched the nasopharyngeal or nasal surface, it was fully rotated clockwise and counterclockwise and placed immediately into a sterile tube. All the samples were stored at − 80 °C until further processing.

### DNA extraction and 16S rRNA gene amplification

DNA extraction was performed via an E.Z.N.A™ MagBind Soil DNA Kit (Omega, M5635-02, USA) according to the manufacturer’s instructions. The concentration of DNA was measured via a Qubit 4.0 (Thermo, USA) to ensure that adequate amounts of high-quality genomic DNA had been extracted.

### 16S rRNA gene amplification by PCR

The PCR was started immediately after DNA extraction. Amplification of the hypervariable region V3–V4 from the 16S rRNA gene was carried out. The 16S rRNA V3–V4 amplicon was conducted via 2 × Hieff^®^ Robust PCR Master Mix (Yeasen, 10105ES03, China). Two universal bacterial 16S rRNA gene amplicon PCR primers [polyacrylamide gel electrophoresis (PAGE)-purified] were used: an amplicon PCR forward primer (CCTACGGGNGGCWGCAG) and an amplicon PCR reverse primer (GACTACHVGGGTATCTAATCC). The reaction mixture was composed of 2 µL of microbial DNA (10 ng/µL), 1 µL of amplicon PCR forward primer (10 µM), 1 µL of amplicon PCR reverse primer (10 µM), and 2 × Hieff® Robust PCR Master Mix (Yeasen, 10105ES03, China) (for a total volume of 30 µL). The plate was sealed, and PCR was performed in a thermal instrument (Applied Biosystems 9700, USA) via the following program: one cycle of denaturation at 95 °C for 3 minutes; five cycles of denaturation at 95 °C for 30 seconds, annealing at 45 °C for 30 seconds, and elongation at 72 °C for 30 seconds; 20 cycles of denaturation at 95 °C for 30 seconds, annealing at 55 °C for 30 seconds, and elongation at 72 °C for 30 seconds; and a final extension at 72 °C for 5 minutes. The PCR products were checked via electrophoresis in 2% (weight/volume) agarose gels in Tris, boric acid, ethylenediamine tetraacetic acid (EDTA) (TBE) buffer, stained with ethidium bromide (EB) and visualized under ultraviolet (UV) light.

### Processing of bacterial 16S rRNA sequencing data and statistical analysis

**Fig. 1:**
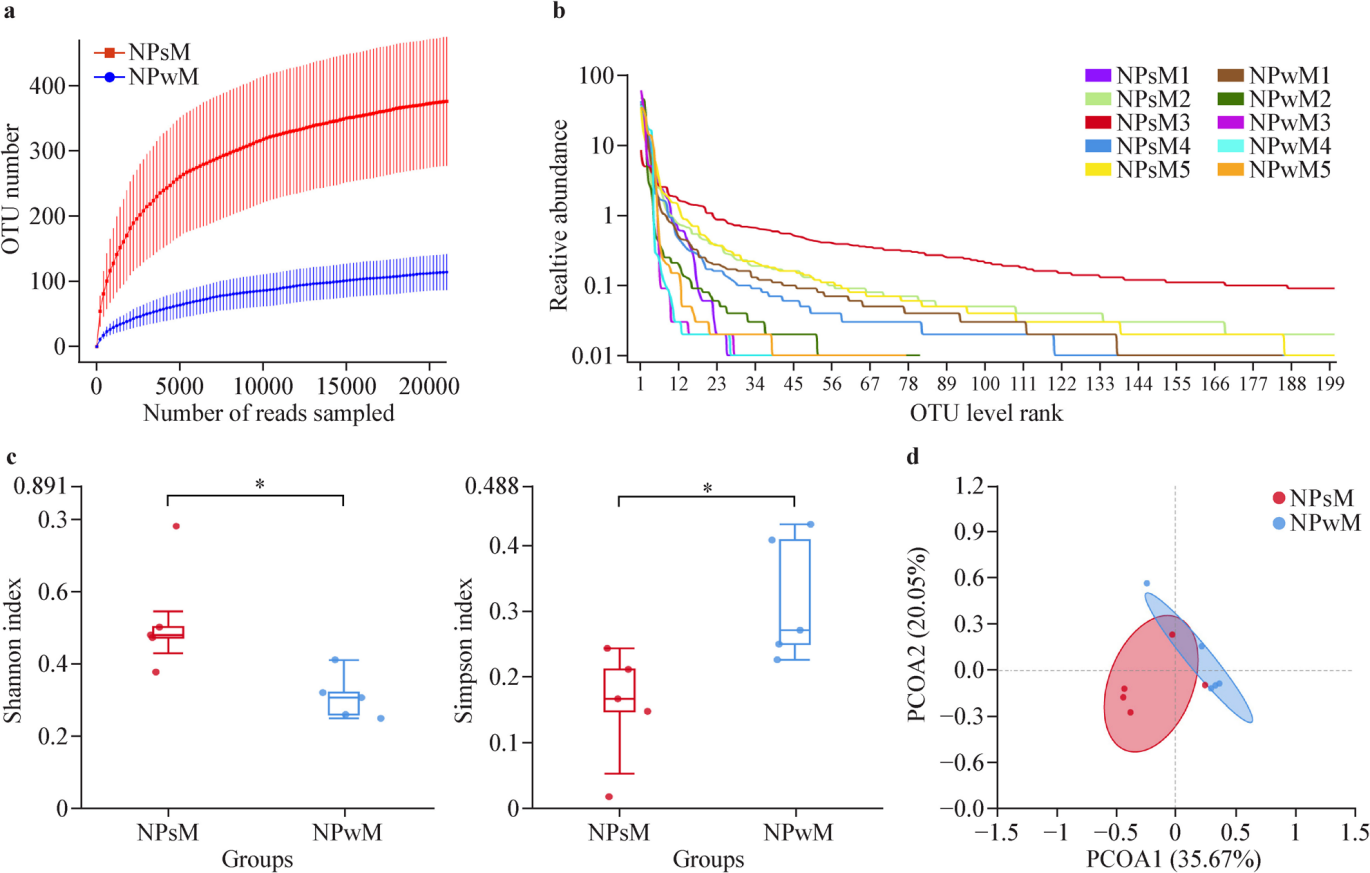
Nasopharyngeal microbiota diversity analysis of NPsM and NPwM (before and after mask wearing) children. **a** Richness dilution curves. For each group, the average values of the α diversity indices with 95% confidence intervals were reported at different sequencing depths. **b** Rank‒abundance curves showing the richness and evenness of the microbiota in each group. The flatter the curve is, the more evenly distributed the microbiota and the greater the microbiota diversity; the wider the horizontal axis of the curve is, the greater the microbiota richness. **c** Box plots showing α diversity estimators (Shannon index and Simpson index) measured for each group. **d** PCOA plot of bacterial β diversity based on weighted UniFrac distances according to individual status. The numbers in parentheses represent the percentage of the total variance explained by the principal coordinates. A *P* value ≤ 0.05 was considered to indicate statistical significance. *P < 0.05. *NPsM* nasopharyngeal samples without mask wearing, *NPwM* nasopharyngeal samples with mask wearing during the pandemic, *OTU* operational taxonomic units

**Fig. 2:**
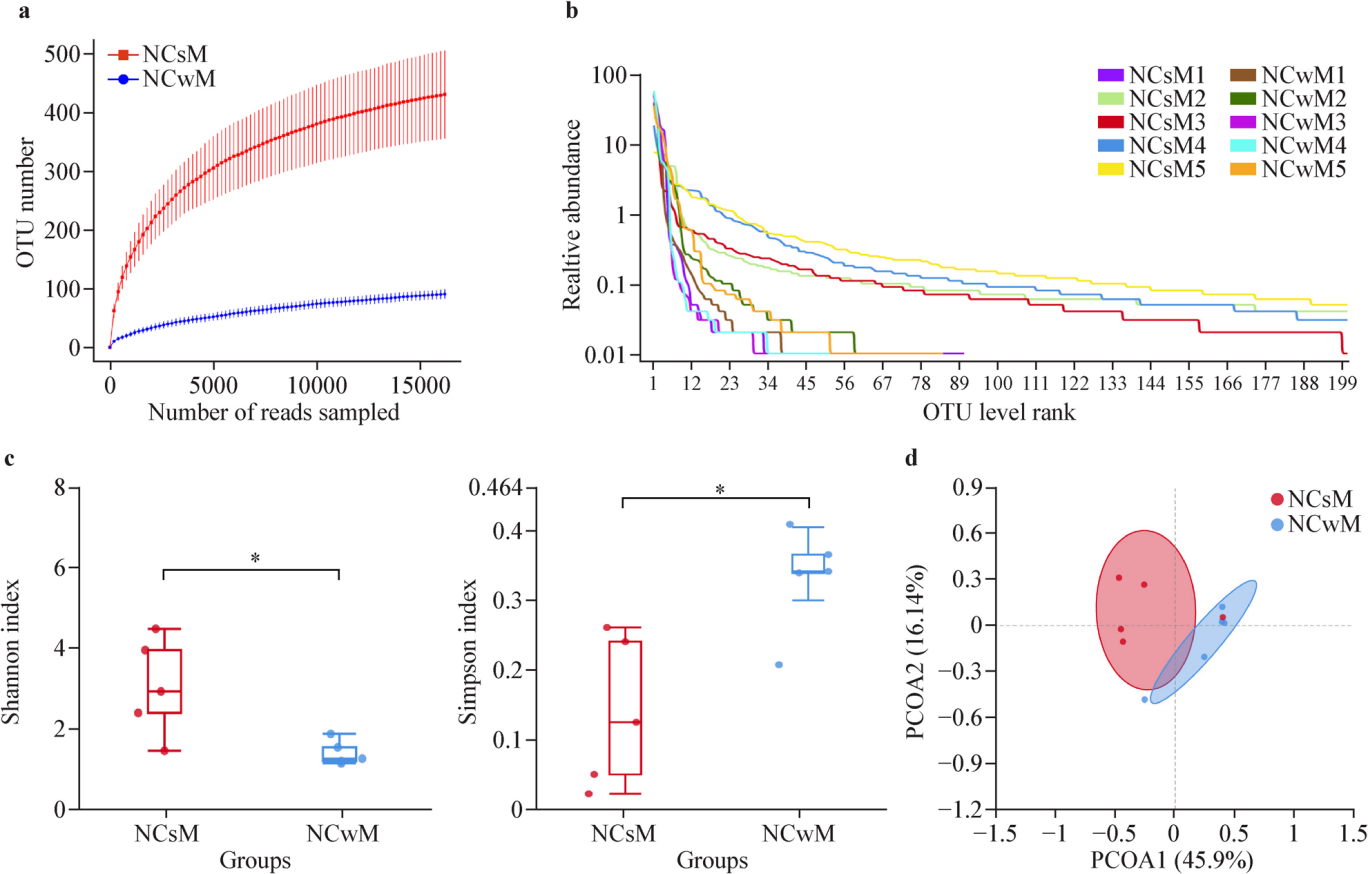
Nasal microbiota diversity analysis of NCsM and NCwM (before and after mask wearing) children. **a** Richness dilution curves. For each group, the average values of the α diversity indices with 95% confidence intervals were reported at different sequencing depths. **b** Rank‒abundance curves showing the richness and evenness of the microbiota in each group. **c** Box plots showing α diversity estimators (Shannon index and Simpson index) measured for each group. **d** PCOA plot of bacterial β diversity based on weighted UniFrac distances according to individual status. The numbers in parentheses represent the percentage of the total variance explained by the principal coordinates. A *P* value ≤ 0.05 was considered to indicate statistical significance. *P < 0.05. *NCsM* nasal samples without mask wearing before the COVID-19 pandemic, *NCwM* nasal samples with mask wearing during the pandemic, *OTU* operational taxonomic units

**Fig. 3:**
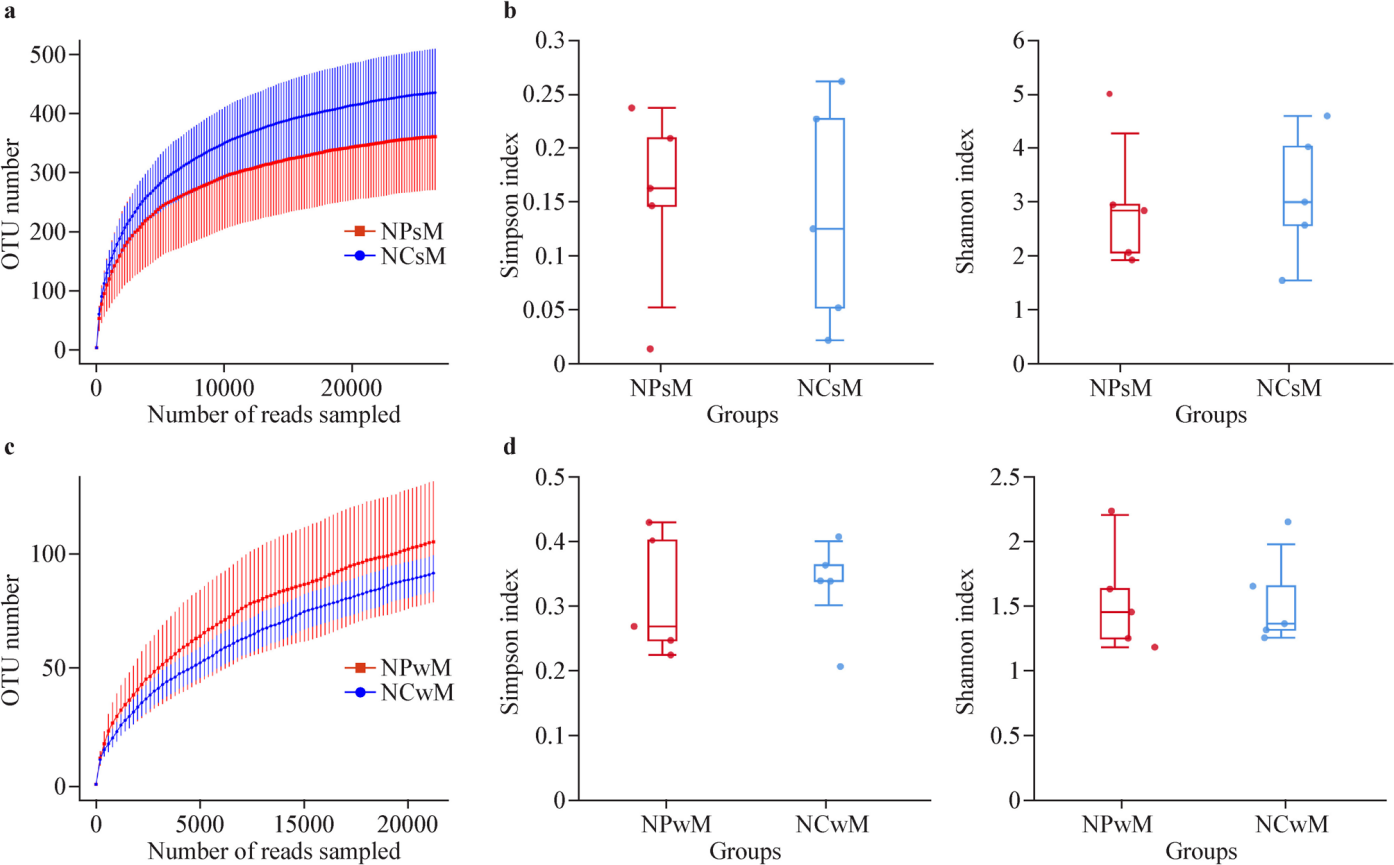
Nasopharynx and nasal microbiota diversity analysis among children before and after mask wearing. **a** Richness dilution curves. For the NPsM and NCsM groups, the average values of the α diversity indices with 95% confidence intervals were reported at different sequencing depths. **b** Box plots showing the α diversity estimators (Shannon index and Simpson index) measured for the NPsM and NCsM groups. **c** Richness dilution curves. For the NPwM and NCwM groups, the average values of the α diversity indices with 95% confidence intervals were reported at different sequencing depths. **d** Box plots showing the α diversity estimators (Shannon index and Simpson index) measured for the NPwM and NCwM groups. A *P* value ≤ 0.05 was considered to indicate statistical significance. *NPsM* nasopharyngeal samples without mask wearing, *NPwM* nasopharyngeal samples with mask wearing during the pandemic, *NCsM* nasal samples without mask wearing before the COVID-19 pandemic, *NCwM* nasal samples with mask wearing during the pandemic, *OTU* operational taxonomic units

Multiple group comparisons of age data were made via analysis of variance (ANOVA), and the chi-square test was used for analysis of sex among groups. Sequencing was performed via the Illumina MiSeq system (Illumina MiSeq, USA) according to the manufacturer’s instructions. The samples were rarefied to a total of 45,353 reads (nasopharyngeal samples) and 50,727 reads (nasal samples), in agreement with the rarefaction curves computed for each diversity index considered (Figs. 1a, 2a, 3a, c). To assess sample adequacy, rarefaction curves of the observed numbers of operational taxonomic units (OTUs) were constructed, and all α diversity indices were calculated via Shannon and Simpson indices with Mothur software (version 3.8.31). The OTU rarefaction curve and rank abundance curves were plotted in R (version 3.6.0). Analyses of microbiota richness, diversity and composition and differences were performed. To estimate the diversity of the microbial community of the sample, we compared the diversity via t test. β diversity was used to evaluate differences in the microbiome among samples and was combined with principal coordinate analysis (PCOA). The analysis was visualized via the R vegan package (version 2.5-6), and finally, the inter-sample distances were presented as scatterplots. Difference comparison was used to identify features with significantly different abundances between groups via STAMP (version 2.1.3) and LefSe (version 1.1.0). Comparisons of the abundance of different microbiota at the phylum and genus levels in the two groups of samples were made via Welch’s *t* test.

### Ethical approval

The study was approved by the ethics committee of Xinhua Hospital, Shanghai Jiao Tong University School of Medicine (No. XHEC-NSFC-2020-045). Informed consent to participate in the study was obtained from the parents of the children.

## Results

A total of 10 pediatric patients without adenoidal hypertrophy provided 20 samples, of which five samples were assigned to the nasopharyngeal samples without mask wearing (NPsM) group, five samples were assigned to the nasal samples without mask wearing before the COVID-19 pandemic (NCsM) group, five samples were assigned to the nasopharyngeal samples with mask wearing during the pandemic (NPwM) group, and five samples were assigned to the nasal samples with mask wearing during the pandemic (NCwM) group. No statistically significant differences in sex or age were found among the groups. All 20 samples were subjected to 16S rRNA gene-based microbiota analysis and were eligible for subsequent bioinformatic downstream analysis. From a total of 807,161 sequences passing the filtering bioinformatics processes (mean ± SD, 40,358.05 ± 16,147.41/samples), 1837 OTUs were identified.

### Alterations in the richness and diversity of the nasopharyngeal and nasal microbiota in children before and during COVID-19

With respect to α diversity, the rank‒abundance curves revealed that most of the curves in the NPwM group (except for NPwM1) were steep, indicating fewer species and lower diversity in the NPwM group. In contrast, most of the curves in the NPsM group (except for NPsM1) were wider than those in the NPwM group were, indicating that the richness of the colonies was greater in the NPsM group (Fig. 1b). With respect to the NCwM and NCsM groups, most of the curves in the NCwM group were steep, indicating fewer species and lower diversity in the NCwM group. In contrast, most of the curves in the NCsM group (except for those in the NCsM1 group) were wider than those in the NCwM group were, indicating that the richness of the colonies was greater in the NCsM group (Fig. 2b). These results indicated that the diversity and richness of the microbiota in the nasopharynx of children without adenoid hypertrophy were lower after mask wearing.

Furthermore, significant differences were found between the NPsM and NPwM groups according to the Shannon and Simpson indices (for the Shannon index, *P* = 0.033; for the Simpson index, *P* = 0.024; Fig. 1c) and between the NCsM and NCwM groups (for the Shannon index, *P* = 0.019; for the Simpson index, *P* = 0.013; Fig. 2c), demonstrating that there was a difference in the diversity of nasopharyngeal and nasal microbiota in children without adenoid hypertrophy before and after mask wearing.

With respect to β diversity, principal coordinate analysis (PCoA) was used to determine statistically significant differences in the weighted UniFrac distance across all the different phenotypes (weighted UniFrac, *P* = 0.001). The results revealed that there were differences in the overall distances, indicating that the compositional structures of the colonies were not identical. Within the groups, most of the distances among the samples in the NPwM and NCwM groups were small and relatively centralized, indicating that the interindividual differences in the structure of the nasopharyngeal and nasal colony communities were relatively small during the pandemic. The distances among the samples in the NPsM and NCsM groups were relatively discrete, indicating that the interindividual differences in the structure of the nasopharyngeal and nasal colonies were more pronounced before the pandemic (Figs. 1d, 2d).

### Comparison of the nasopharynx and nasal microbiota among children without adenoid hypertrophy before and after mask wearing

With respect to α diversity, no statistically significant difference was found between the NPsM and NCsM groups according to the Shannon and Simpson indices, demonstrating that there was no difference in the diversity of the nasopharyngeal and nasal microbiota in children before mask wearing (for the Shannon index, *P* = 0.81; for the Simpson index, *P* = 0.80; Fig. 3b). Moreover, no statistically significant difference was found between the NPwM and NCwM groups, demonstrating that there was no difference in the diversity of the nasopharyngeal and nasal microbiotas in children after mask wearing (for the Shannon index, *P* = 0.88; for the Simpson index, *P* = 0.76; Fig. 3d).

### Analysis of the composition and differences in the nasopharyngeal microbiota of children without adenoid hypertrophy before and during the pandemic

A total of 26 different phyla and 140 genera were detected across all the samples, of which only six (23%) phyla and 26 (19%) genera presented a mean relative abundance ≥ 1% in at least one considered phenotype. *Firmicutes* was the most abundant phylum in all the groups, followed by *Proteobacteria*, *Actinobacteria*, *Bacteroidetes*, *Fusobacteria*, and *Spirochaetes* to different extents in all the samples. *Spirochaetes* were present only in the NPsM group and not in the NPwM group (Supplementary Fig. 1a, left).

At the genus level, a more variable distribution of microbiota was demonstrated, with examples showing *Pseudomonas* (23%) and *Corynebacterium* (14%) as the two most abundant genera in the NPsM group, whereas *Corynebacterium* (29%) and *Moraxella* (24%) had the highest mean relative abundances in the NPwM group (Supplementary Fig. 1a right).

The collinearity diagram is a visual circle diagram that describes the correspondence between samples, species, and functions; it reflects not only the proportion of dominant species in each sample but also the distribution of each dominant species among different samples. We found that at the genus level, there were large differences in the number of colonies among NPsM samples and relatively small differences among the NPwM samples (Supplementary Fig. 1b).

Welch’s *t* test comparing the differences between the two groups for each species with the lowest percentage of phylum-level sequence numbers revealed that the microbiota of the NPsM and NPwM groups differed significantly in terms of *Cyanobacteria/Chloroplast* (from 0.44% before the pandemic to 0.03% during the pandemic) and *Bacteroidetes* (from 9% before the pandemic to 0.07% during the pandemic) (Supplementary Fig. 1c). At the genus level, the microbiota of the NPsM and NPwM groups differed significantly in terms of the abundance of *Streptophyta*, *Agrobacterium*, *Acidovorax*, *Aeromonas*, *Ligilactobacillus*, *Weissella*, *Prevotella*, *Nitrilliruptoria*, *Asticcacaulis*, *Brevundimonas*, *Roseibium*, *Rhodococcus*, *Nesterenkonia*, *Porphyrobacter*, *Bacillaceae 1*, *Pasteurellaceae*, *Sphingomonas*, *Metabacillus*, *Paenibacillus*, *Peptococcus*, *Phocaeicola*, *Peptostreptococcaceae*, and *Devosia* (Supplementary Fig. 1c). There was no significant difference in the abundance of *Pseudochelatococcus* or *Cellulomonas* between the nasopharyngeal microbiota of the children in the two groups (Supplementary Fig. 1d).

### Analysis of the composition and differences in the nasal microbiota of children without adenoid hypertrophy before and during the pandemic

A total of 24 different phyla and 168 genera were detected across all the samples, of which only six (25%) phyla and 26 (15%) genera presented a mean relative abundance ≥ 1% in at least one considered phenotype. *Proteobacteria* was the most abundant phylum in all the groups, followed by *Firmicutes*, *Actinobacteria*, *Bacteroidetes*, *Cyanobacteria/Chloroplast*, and *Euryarchaeota* to different extents in all the samples. *Euryarchaeota* was present only in the NCsM group and not in the NCwM group (Supplementary Fig. 2a, left).

At the genus level, a more variable distribution of microbiota was demonstrated, with *Pseudomonas* (22%) and *Corynebacterium* (10%) being the two most abundant genera in the NCsM group, whereas *Moraxella* (33%) and *Corynebacterium* (26%) were the most abundant in the NCwM group (Supplementary Fig. 2a, right).

According to the collinearity diagram, there were large differences in the colonies among NCsM samples and relatively small differences among the NCwM samples at the genus level (Supplementary Fig. 2b). Welch’s *t* test, which compares the differences between the two groups for each species with the lowest percentage of phylum-level sequence numbers, revealed that the microbiota of the NCsM and NCwM groups differed significantly in terms of *Planctomycetes* (from 0.16% before the pandemic to 0% during the pandemic, Supplementary Fig. 2c). At the genus level, the microbiota of the NCsM and NCwM groups differed significantly in terms of *Sphingobium*, *Rhizobiales*, *Agrobacterium*, *Lachnospiraceae*, *Fusicatenibacter*, *Ligilactobacillus*, *Acidovorax*, *Dorea*, *Intestinimonas*, *Acetobacteraceae*, *Olsenella*, *Lachnospiracea incertae sedis*, *Enhydrobacter*, *Sphingomonas*, *Methanoculleus*, *Gp3*, and *Mitsuokella*. There was no significant difference in the nasal microbiota of the children in the two groups for *Devosia*, *Enterococcus*, *Zea*, *Lactobacillus*, *Bacteroidales*, *Brevibacterium*, *Sarcina*, or *Methylobacterium* (Supplementary Fig. 2d)*.*

## Discussion

This study is aimed to investigate the microbiota of the nasopharynx and nasal cavity in healthy children without adenoid hypertrophy before and during the COVID-19 pandemic. Lower Shannon indices, higher Simpson indices, and more clustered samples in the unweighted UniFrac distance matrix all indicated lower microbiota diversity during COVID-19. This was a very understandable result because limiting contact with infected individuals through physical distancing measures such as wearing masks decreased transmissibility in both clinical and laboratory settings by limiting the spread of infectious respiratory particles [16]. In China, in addition to mask wearing, isolation procedures such as increased online education and reduced preschool childcare and elementary school attendance are strictly adopted, which are likely to decrease microbiota diversity in children.

Our study suggests that the nasopharyngeal microenvironment is correlated with the nasal cavity, while we detected changes in the composition of the microbiota in the nasopharynx and nasal cavity in children after mask wearing. Our results are in agreement with those of a previous study in which *Firmicutes* and *Proteobacteria* were reported as the most abundant bacterial groups in healthy children [25–27]. Another study during the pandemic reported that the five main phyla in the nostrils were *Firmicutes*, *Proteobacteria*, *Bacteroidetes*, *Actinobacteria*, and *Fusobacteria* [28], and our study further revealed that *Firmicutes* (36%) was the most abundant phylum in the nasopharyngeal microbiota and that *Proteobacteria* (46%) was the most abundant phylum in the nasal microbiota. *Bacteroidetes*, in conjunction with *Actinobacteria* and *Firmicutes*, form a healthy lung microbial environment [29, 30]. *Bacteroidetes* are more common in healthy individuals than in asthmatic individuals [31]. In a mouse model of colitis, a conserved enzyme of the *Bacteroidetes* phylum protected against intestinal inflammation [32]. Researchers have reported that *Bacteroidetes* is one of the major phyla in the adenoids, tonsils, oropharynx, and nostrils, but is least abundant in the nostrils [28]. Another study revealed that antibiotic exposure was associated with a decreased abundance of *Bacteroidetes* in infants [33]. *Cyanobacteria/chloroplasts* can be found in the gut microbiome [34] and thrive in a wide range of places, including rocks and soils [35]. Analyses of 16S rRNA sequences at the phylum level also revealed that *Cyanobacteria/Chloroplast* is one of the most common phyla in root and leaf samples [35]. *Planctomycetes* are known to inhabit the surfaces of aquatic habitats, such as macroalgae [36], plants [37], and crustaceans [38]. Here, we observed a decrease in the phyla *Bacteroidetes*, *Cyanobacteria/Chloroplast*, and *Planctomycetes* during the pandemic, indicating that mask wearing and other physical isolation measures, such as alienation from nature and reduced outdoor activities, could cause microbiota dysbiosis.

According to some previous studies [39, 40], *Moraxella* and *Corynebacterium* represented the two most abundant genera in the nasopharynx and nasal cavity in healthy individuals, which supported our results. Researchers have reported that *Moraxella* could be active against *Streptococcus pneumoniae* [41] and was associated with a lower frequency of upper respiratory tract infections [42]. Interestingly, despite its beneficial properties, *Moraxella* has also been associated with opportunistic infections in the upper respiratory tract and an increased risk of acute sinusitis [43, 44]. We believe that *Moraxella* should be viewed as a common component of the nasopharyngeal and nasal microbiota rather than a pathogen. Its biological behavior can be complex, and further studies focused on its function are needed. It has also been reported that *Corynebacterium* can inhibit the growth or virulence of *Staphylococcus aureus* [9] and can lower the frequency of upper respiratory tract infections [42] and rhinosinusitis [45, 46]. It may help maintain a balanced microbiota by eradicating pathogenic bacteria and preventing upper respiratory diseases in healthy children. Conversely, the enrichment of *Pseudomonas* in diseased individuals indicates its potential pathogenic role in human respiratory tract infections [47, 48]. In our study, *Pseudomonas* was dominant before the pandemic but dramatically decreased during the pandemic from 23 to 0.01% in the nasopharynx and decreased from 22 to 1% in the nasal cavity. We suspect that the decreased abundance of *Pseudomonas* during the COVID-19 pandemic prevented children from developing respiratory diseases.

Decreased richness and diversity, as well as changes in these microbiota can strongly influence the prevalence of respiratory infections. We followed the study sample and found that all the children were infected with COVID-19 after the pandemic was declared. We also noted more frequent use of antibiotics and other drugs during the pandemic to alleviate bacterial infections and gastrointestinal symptoms. This may have caused a significant decrease in abundance and dysbiosis in the aforementioned microbiota, especially in the beneficial bacteria, further dysregulating the bidirectional crosstalk across the gut–lung axis, resulting in hypersensitivity and hyperreactivity to respiratory allergens. Moreover, we have observed a decrease in mask wearing since the pandemic was declared to be under control. The influenza wave in the winter of 2023 was more intense than that in the years prior to the COVID-19 outbreak, as reported by the China Influenza Surveillance Weekly Newspaper. The term “immune debt” was first used in 2021 by French academics, who reported that a protracted period of “low exposure to pathogens” increased the proportion of susceptible individuals and increased the possibility of future outbreaks [49]. We propose that isolation procedures are double-edged swords. The positive collateral effect in the short term is beneficial because it reduces infectious diseases and prevents additional overload of the healthcare system. However, owing to the reduced circulation of microbial agents, a lack of immune stimulation can have negative consequences. This is due to a growing proportion of "susceptible" children and decreased herd immunity in the population. We suppose that some pathogenic microorganisms may take advantage of the opportunity to become dominant in the microbiota, disrupting the dynamic balance of microbial colonization and causing microecological dysfunction. This may result in insufficient nasopharyngeal and nasal mucosal clearance of pathogens, thereby triggering several respiratory tract diseases.

COVID-19-related microbiota investigations offer novel insights into respiratory disease mechanisms, diagnostic and therapeutic advancements, and public health approaches. In preventive medicine, elucidating the relationship between respiratory diseases and microbial colonization in the nasopharynx and nasal cavity is critical for formulating effective public health strategies to safeguard susceptible pediatric populations. This research underscores the imperative for disease prevention and mitigating healthcare system strain through strategies such as increased outdoor exposure (with avoidance of prolonged exposure to air pollution [50]), moderation in cleaning practices, judicious antibiotic use, and mask adherence for vulnerable and infected individuals. For clinical application, the findings from this study can aid in the development of microbiota-based biomarkers for early disease prediction and prognostic assessment. Personalized treatments and targeted interventions, including probiotics and phage-mediated clearance of respiratory pathogens, can be developed. Future interventions may consider implementing precise regulation of the upper respiratory tract microbiota by inhalation or administration of drops, in a similar pattern to how fecal microbiota transplantation (FMT) safely restores the gut microbiota balance and treats both gastrointestinal disorders and extragastrointestinal diseases [29, 51, 52]. In primary healthcare, microbiota management is anticipated to enhance the overall respiratory health of the pediatric population by shifting the focus from disease treatment to disease prevention and health maintenance. Integrating straightforward microbiota testing into routine primary care can facilitate early identification of high-risk populations and individuals exhibiting microbiota dysbiosis. Ongoing primary healthcare reforms and initiatives, such as the establishment of health and wellness centers and programs such as Ayushman Bharat in India, are underway in numerous countries [53–55]. Pandemics are defining novel health challenges of the twenty-first century [56]. This renewed attention to primary healthcare and related reform should be leveraged to engage communities in raising awareness and implementing actions to address the challenges posed by infectious disease outbreaks and epidemics [57].

There were several limitations of our study. First, the study was conducted on a relatively small number of children, and our results need to be confirmed in larger samples. However, it is currently difficult to recruit more children because COVID-19 is no longer a public health emergency; the timing makes this study unique. Second, our study covered only the midterm stage of the pandemic. It is difficult to determine whether there were any dynamic differences in the outcomes during the entire pandemic. Third, although our study did not reveal that the nasopharyngeal microbiota was more abundant than the nasal microbiota was, nasopharyngeal samples collected through the nasal cavity may be contaminated, apparently increasing the nasopharyngeal microbiota. Oral sampling or specific collection tools should be considered. Fourth, comprehensive clinical pathogen identification is limited by the lack of currently available 16S sequence data. To address this, high-fidelity sequencing of full-length 16S rRNA genes or whole-genome shotgun (WGS) sequencing can be employed, utilizing complete gene sequences to increase resolution. Furthermore, alternative microbiome analysis tools, including 18S rRNA gene sequencing and internal transcribed spacer (ITS) region analysis, can supplement 16S rRNA gene sequencing to facilitate fungal identification.

In conclusion, prolonged mask wearing and isolation measures during the COVID-19 pandemic provided us with a rare opportunity to study variations in the nasopharyngeal and nasal microbiota in children. Loss of microbial diversity and microbiota dysbiosis partly explain the ensuing peak of respiratory infectious diseases. Our study provides insights into the biological mechanisms of potential and future respiratory tract diseases in the pediatric population and raises awareness for reestablishing a balance among microbiota populations to prevent or even treat these diseases.

## Supplementary Information

Below is the link to the electronic supplementary material.

Supplementary file1 (DOCX 678 KB)

Supplementary file2 (DOCX 15288 KB)

## Data Availability

All data generated or analysed during this study are included in this published article.
