# Supplementary material for: Characteristics of the microbiota in the nasopharynx and nasal cavity of healthy children before and during the COVID-19 pandemic
Source: World J Pediatr. 2025 Jul 31;21(8):836–45. doi: 10.1007/s12519-025-00953-z (PMC12380981; doi:10.1007/s12519-025-00953-z)
Supplement: Supplementary file 2 — Supplementary file1 (DOCX 678 KB) [file 12519_2025_953_MOESM1_ESM.docx]

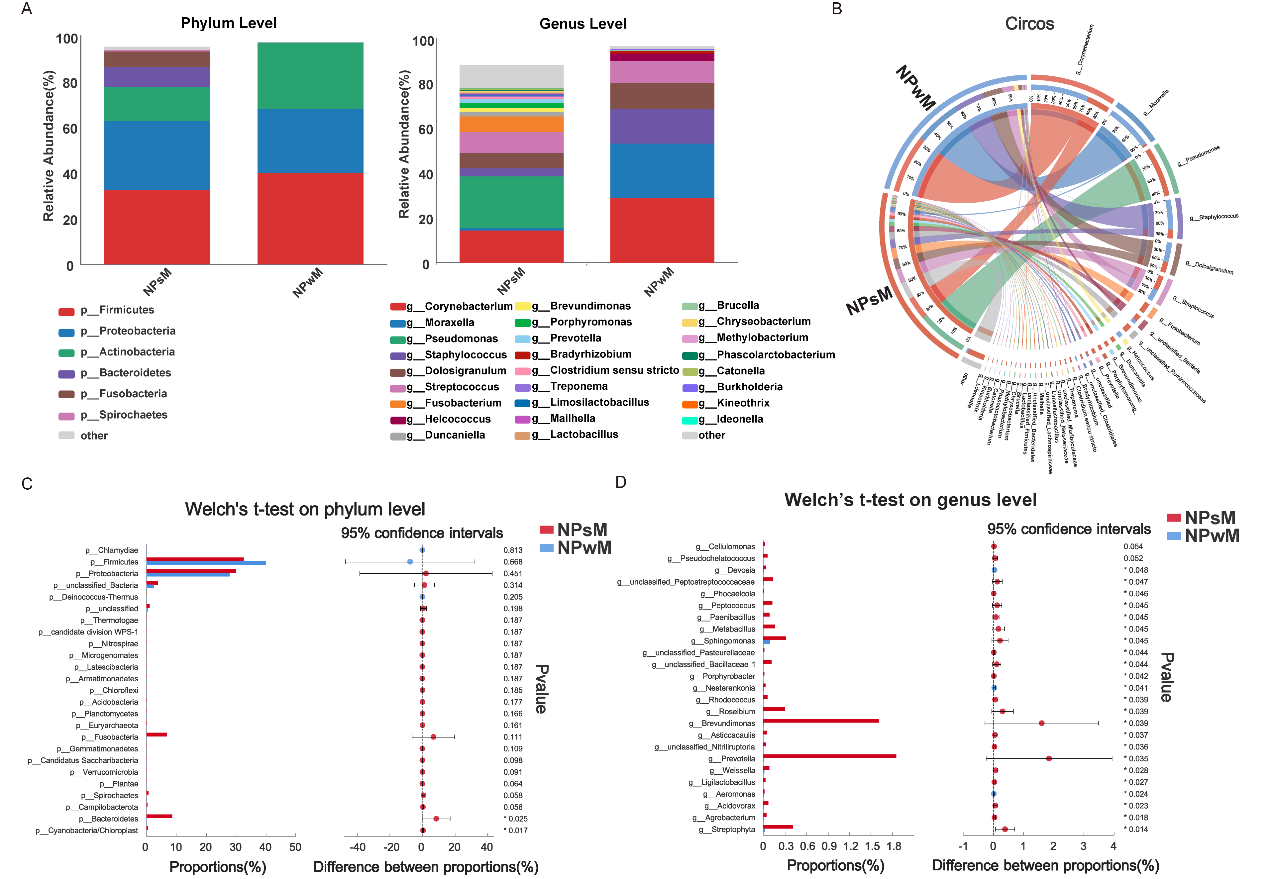


**Supplementary figure 1** Analysis of the composition and differences of the nasopharyngeal microbiota in children without adenoid hypertrophy. a Color-coded bar plots showing the average distribution of bacterial microbiota at the phylum (left) and genus (right) levels across different phenotypes. Only microbiota for which a mean relative abundance = 1% was determined in at least one group were reported in plots. b Chord diagrams showing the composition of each individual community and interindividual differences at the genus level. Species with a mean relative abundance >1% were reported in plots. The right half-circle indicates the microbiota composition of the samples, and the left half-circle indicates the proportional distribution of the species among different samples. c The proportions of the different microbiota at the phylum level in the two groups of samples are shown on the left, the proportion of differences in the abundance of microbiota within the 95% confidence interval is shown in the middle, and *P* values are shown on the right. Only the 25 species with the lowest *P* values are listed. d The proportions of the different microbiota at the genus level in the two groups of samples are shown on the left, the proportion of differences in the abundance of microbiota within the 95% confidence interval is shown in the middle, and *P* values are shown on the right. Only the 25 species with the lowest *P* values are listed. A *P* value ≤ 0.05 was considered to indicate statistical significance. **P* < 0.05.


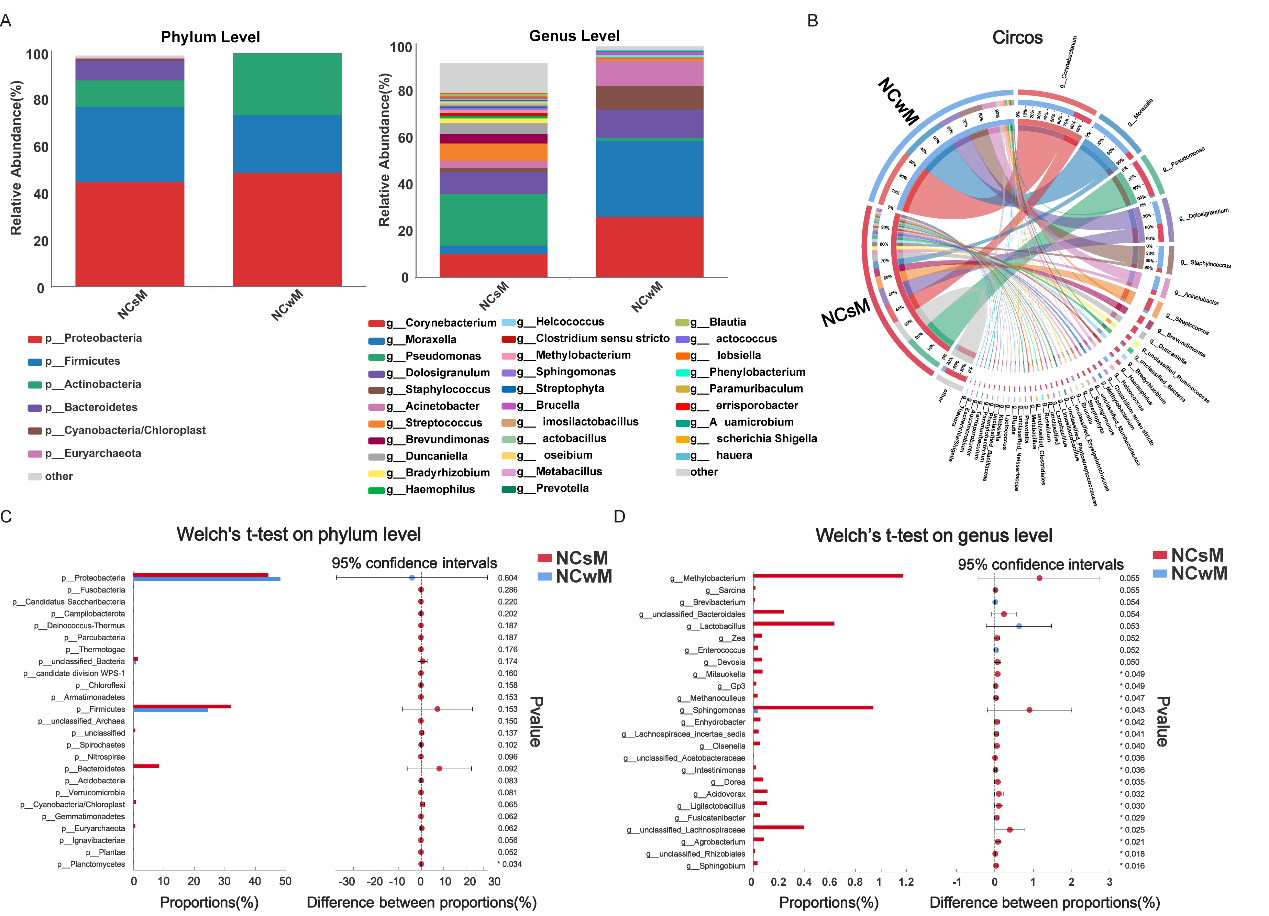


**Supplementary figure 2** Analysis of nasal microbiota composition and differences in children without adenoid hypertrophy. a Color-coded bar plots showing the average distribution of bacterial microbiota at the phylum (left) and genus (right) levels across different phenotypes. Only microbiota for which a mean relative abundance = 1% was determined in at least one group were reported in plots. b Chord diagrams showing the composition of each individual community and interindividual differences at the genus level. Species with a mean relative abundance >1% were reported in plots. The right half-circle indicates the microbiota composition of the samples, and the left half-circle indicates the proportional distribution of the species among different samples. c The proportions of different microbiota at the phylum level in the two groups of samples are shown on the left, the proportion of differences in the abundance of microbiota within the 95% confidence interval is shown in the middle, and *P* values are shown on the right. Only the 25 species with the lowest *P* values are listed. d The proportions of different microbiota at the genus level in the two groups of samples are shown on the left, the proportion of differences in the abundance of microbiota within the 95% confidence interval is shown in the middle, and *P* values are shown on the right. Only the 25 species with the lowest *P* values are listed. A *P* value ≤ 0.05 was considered to indicate statistical significance. **P* < 0.05.
