# Supplementary material for: Characteristics of the microbiota in the nasopharynx and nasal cavity of healthy children before and during the COVID-19 pandemic
Source: World J Pediatr. 2025 Jul 31;21(8):836–45. doi: 10.1007/s12519-025-00953-z (PMC12380981; doi:10.1007/s12519-025-00953-z)
Supplement: Supplementary file 3 — Supplementary file2 (DOCX 15288 KB) [file 12519_2025_953_MOESM2_ESM.docx]

WJP| COVID-19 流行前和流行期间健康儿童鼻咽和鼻腔微生物群的改变


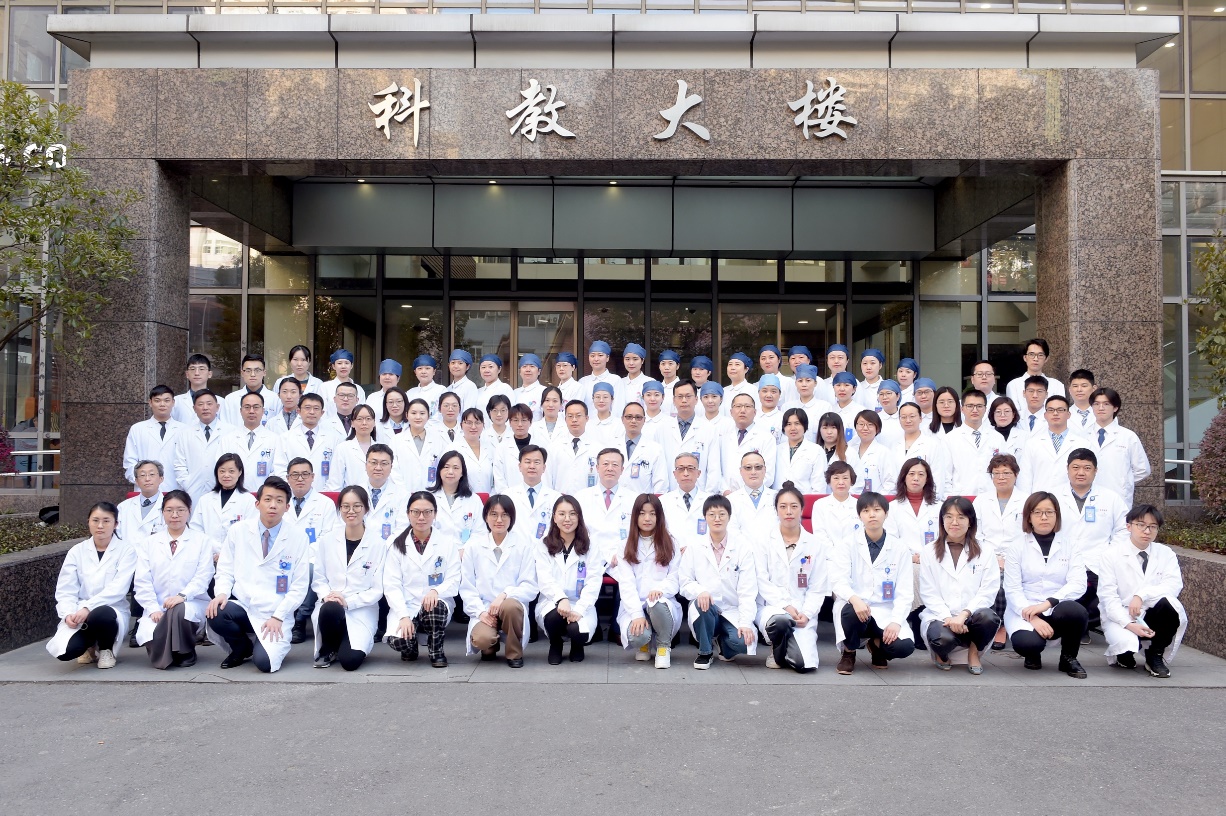

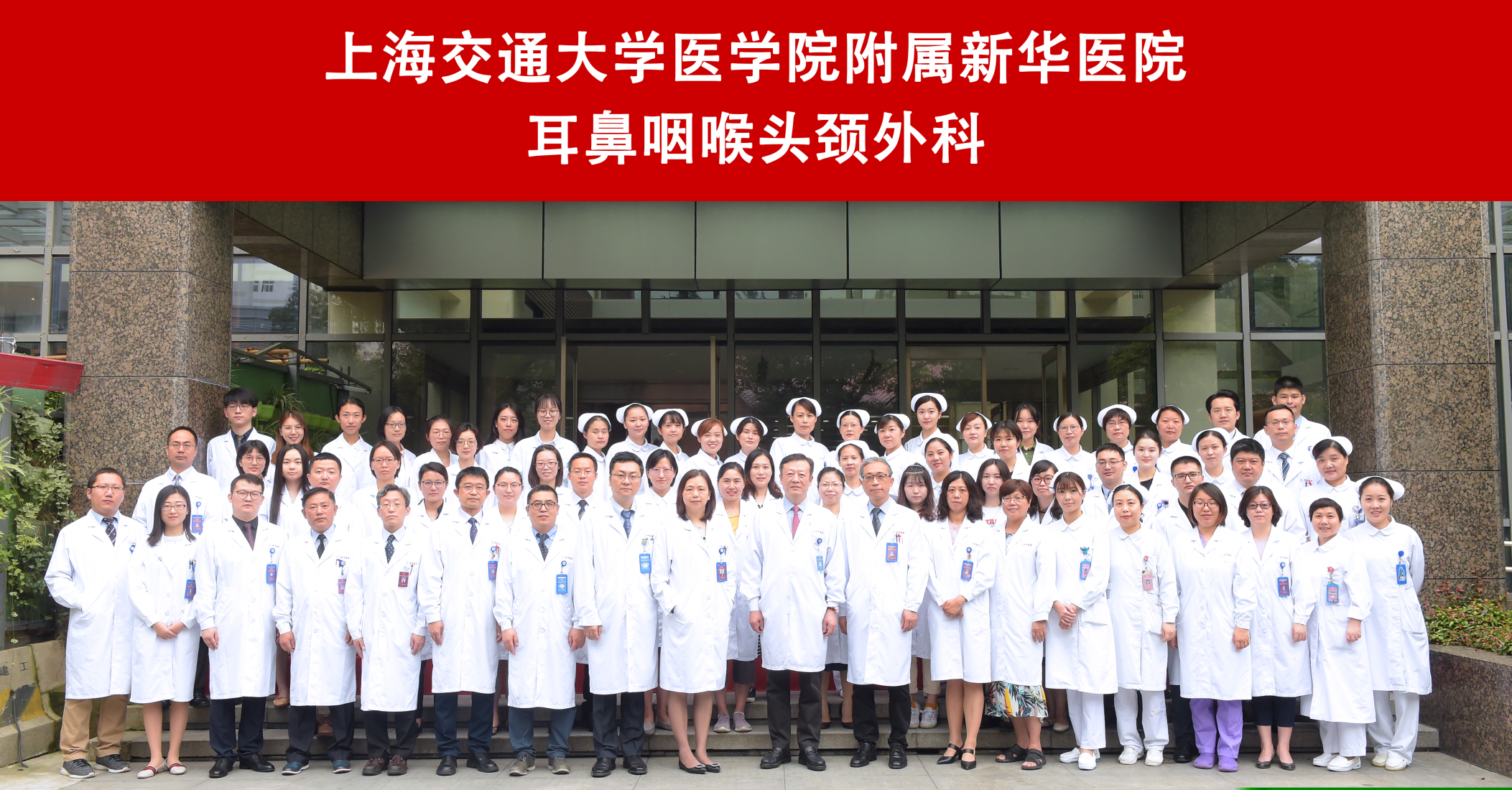


通讯作者简介：


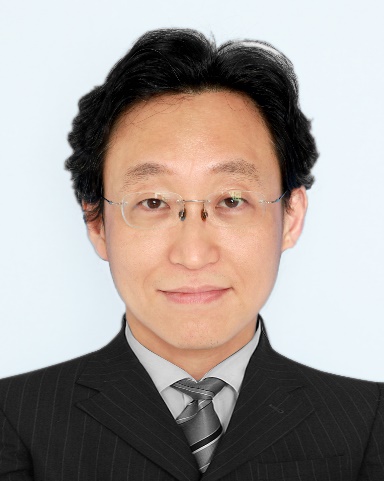


姓 名：魏崴

医学博士、副主任医师

本科毕业于上海医科大学，硕士毕业于复旦大学附属眼耳鼻喉科医院，博士毕业于上海交通大学医学院附属第九人民医院。

上海市医学会耳鼻咽喉头颈外科学分会鼻科学组委员及创伤学组委员，中国中药耳鼻咽喉药物研究专业委员会委员，上海口腔医学会口腔黏膜病专委会委员。

从事耳鼻咽喉头颈外科临床工作三十年，擅长鼻腔鼻窦、口鼻颌面、鼻眼、鼻颅底相关炎症、肿瘤、创伤、畸形等疾患的诊疗，小儿与成人鼾症诊治，过敏性鼻炎与鼻咽癌外科治疗，声带嗓音疾病微创手术。

主要研究方向为上呼吸道炎症与组织器官区域免疫特性，近年来组建腺样体、扁桃体组织样本库，聚焦儿童腺样体、扁桃体肥大病因及机制研究，参与多项国家自然科学基金与省级课题，发表中英文论著30篇，参编著作4部，参译1部；实用新型专利4项。


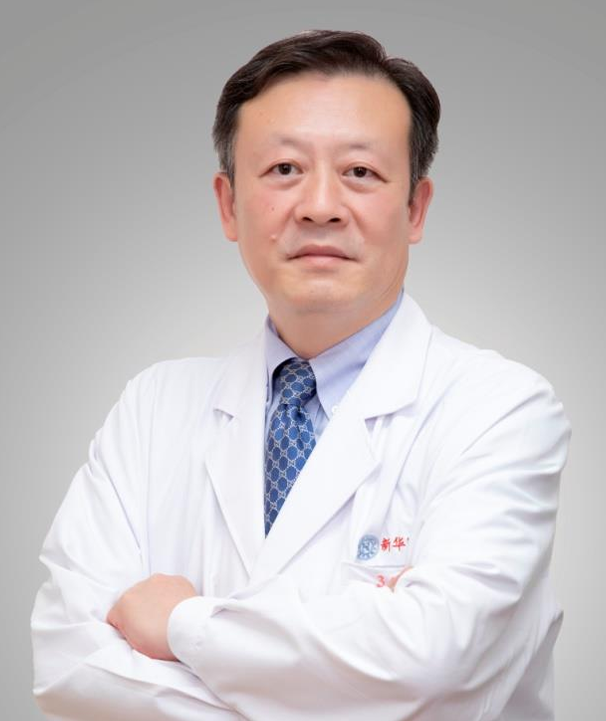


姓 名：杨军

主任医师、上海交通大学致远荣誉计划博士研究生导师，国家临床重点专科上海交通大学医学院附属新华医院耳鼻咽喉头颈外科主任，上海交通大学医学院耳鼻咽喉科学系副主任，外科党总支书记。上海市优秀学科带头人，上海东方英才计划领军人才。擅长成人和儿童人工耳蜗植入，双侧重度极重度感音神经性耳聋MDT评估，各种眩晕疾病的诊断与鉴别，梅尼埃病的综合治疗、根据眩晕程度和听力水平采取的各种手术治疗及前庭康复，颞骨胆脂瘤、听神经瘤、颈静脉孔区肿瘤、外耳道癌等颞骨侧颅底良恶性肿瘤的手术治疗，外伤性面瘫、面神经瘤、面神经炎等面神经疾病的外科治疗，慢性中耳炎、中耳胆脂瘤等中耳疾病的鼓室成形手术，各类听力障碍疾病的干预、听觉言语康复，咽鼓管功能不良的球囊扩张手术。担任上海医师协会理事、上海医师协会耳鼻咽喉科医师分会会长、中国医师协会耳鼻咽喉头颈外科医师分会常委、中国优生科学协会听觉前庭医学分会主任委员、第四届“仁心医者·上海市杰出专科医师”、Barany协会会员，Barany教育委员会委员。《眩晕诊断学》、《眩晕内科诊治和前庭康复》、《眩晕外科手术图谱》丛书总主编，《颞骨侧颅底解剖与手术图谱》、《耳源性眩晕自助手册》、《听力疾病自助手册》主编。《Frontiers in Neurology》杂志副主编、《Acta Oto-Laryngologica》杂志编委，《中华耳鼻咽喉头颈外科杂志》等九种中文杂志编委。国家重点研发计划重点专项首席科学家；主持国家重点研发、国家自然科学基金重点/面上项目8项，获得国家科技进步二等奖1项、上海市医学科技二等奖2项。担任第八届梅尼埃病及内耳疾病国际论坛大会主席。


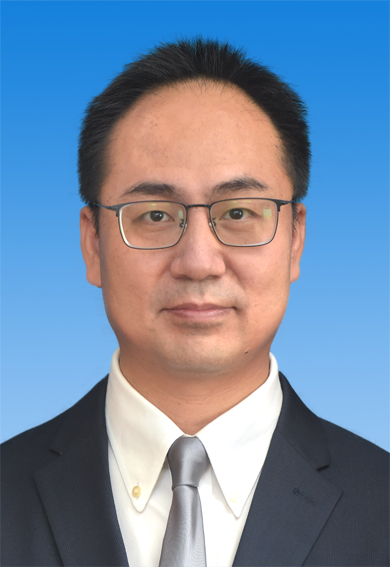


姓 名：何景春

主任医师，医学博士，博士后，博士研究生导师，颞骨侧颅底解剖中心主任。上海交通大学医学博士、美国纽约州立大学博士后，意大利著名Gruppo Otologico耳科及侧颅底外科中心专科培训医师，从事本专业20余年。第二届上海市十佳优秀青年医师，院优青人才计划获得者，中国中西医结合耳鼻咽喉委员会眩晕病专家委员会、中国老年医学学会耳科学分会等多个学会委员，主编专著2部，副主编专著2部，主译专著1部，主持国自然面上项目3项，发表相关论著30余篇，作为主要完成人荣获上海市科技进步一等奖1项。专业特长：耳显微外科、侧颅底外科，擅长成人及小儿各类中耳病变、耳聋、眩晕、面神经疾病、听神经瘤、颅底肿瘤、腮腺肿瘤、人工耳蜗植入，以及鼻内窥镜、儿童鼾病等耳鼻咽喉科内镜微创治疗。

前言

今天小编为大家带来上海交通大学医学院附属新华医院梁敏等作者近期发表在世界儿科杂志（WJP）的文章，了解COVID-19 流行前和流行期间健康儿童鼻咽和鼻腔微生物群的特征和变化。

COVID-19 流行前和流行期间健康儿童鼻咽和鼻腔微生物群的特征

Characteristics of the microbiota in the nasopharynx and nasal cavity of healthy children before and during the COVID-19 pandemic

研究背景

鼻咽和鼻腔中的微生物定植对儿童起着防御作用，这些部位的微生物群失调与儿科众多疾病有关。

2020 年 1 月 30 日，世界卫生组织宣布 COVID-19 为全球紧急卫生事件。在疫情流行期间使用口罩可能有助于减少呼吸道病毒的传播，降低呼吸道感染的概率。然而，人们对 COVID-19流行之前和流行期间健康儿童鼻咽和鼻腔中微生物群定植是否发生变化知之甚少。本研究旨在识别并比较COVID-19 流行之前和流行期间健康儿童鼻咽和鼻腔微生物群的情况。

研究方法

本研究为回顾性研究，研究对象为4-8岁没有被诊断腺样体肥大的健康儿童，他们于2019年2月至2021年8月在上海交通大学医学院附属新华医院耳鼻咽喉-头颈外科接受内镜检查，用咽拭子分别采集鼻咽部和鼻腔表面，将标本分为疫情前未佩戴口罩鼻咽部组、疫情前未佩戴口罩鼻腔组、疫情期间佩戴口罩鼻咽部组和疫情期间佩戴口罩鼻腔组。采用基于 16S rRNA测序技术来描述和分析鼻咽和鼻腔微生物群的变化，以确定戴口罩等隔离措施是否会影响微生物生态。

纳入标准：

①腺样体不肥大

②年龄4-8岁之间

③监护人签署知情同意书

排除标准：

①有腺样体肥大

②目前患有中耳炎、过敏性鼻炎、急性/慢性鼻窦炎或急性呼吸道感染

③两个月内有全身或局部使用糖皮质激素、抗生素或生理盐水鼻腔冲洗史

④有扁桃体切除术、腺样体切除术或鼓膜置管术史

研究结果

研究回顾了 10名健康儿童共10分鼻咽部标本和10份鼻腔标本，所有 20份样本都进行了基于 16S rRNA 基因的微生物群分析，并成功进行了后续的生物信息下游分析。

主要观察指标：

COVID-19 流行期间鼻咽和鼻腔微生物群的丰富度和多样性与疫情流行前相比有所下降（*P* < 0.05，Figure 1A and 1B）。无论是在疫情流行前还是在疫情流行期间，厚壁菌和变形菌分别是鼻咽和鼻腔微生物群中最丰富的菌门（Figure 1C and 1D）。


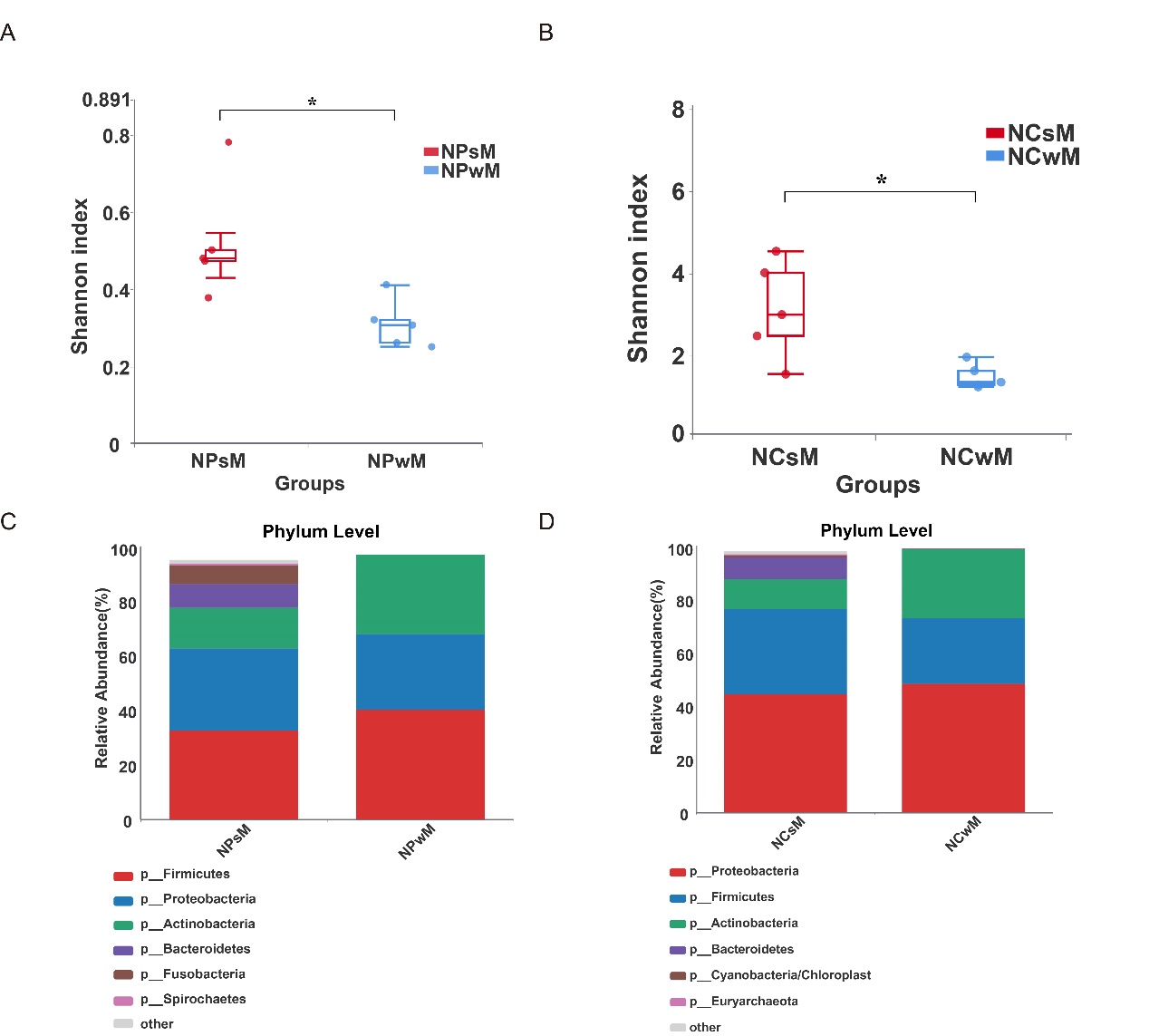


次要观察指标：

在 COVID-19流行期间，棒状杆菌和莫拉菌是鼻咽和鼻腔微生物群中的优势菌属，而在疫情流行前，假单胞菌和棒状杆菌是优势菌属（Figure 2A and 2B）。在COVID-19流行期间，鼻咽部蓝藻/叶绿体和类杆菌属以及鼻腔浮霉菌的微生物定植与疫情前相比有显著差异（*P* < 0.05，Figure 2C and 2D）。


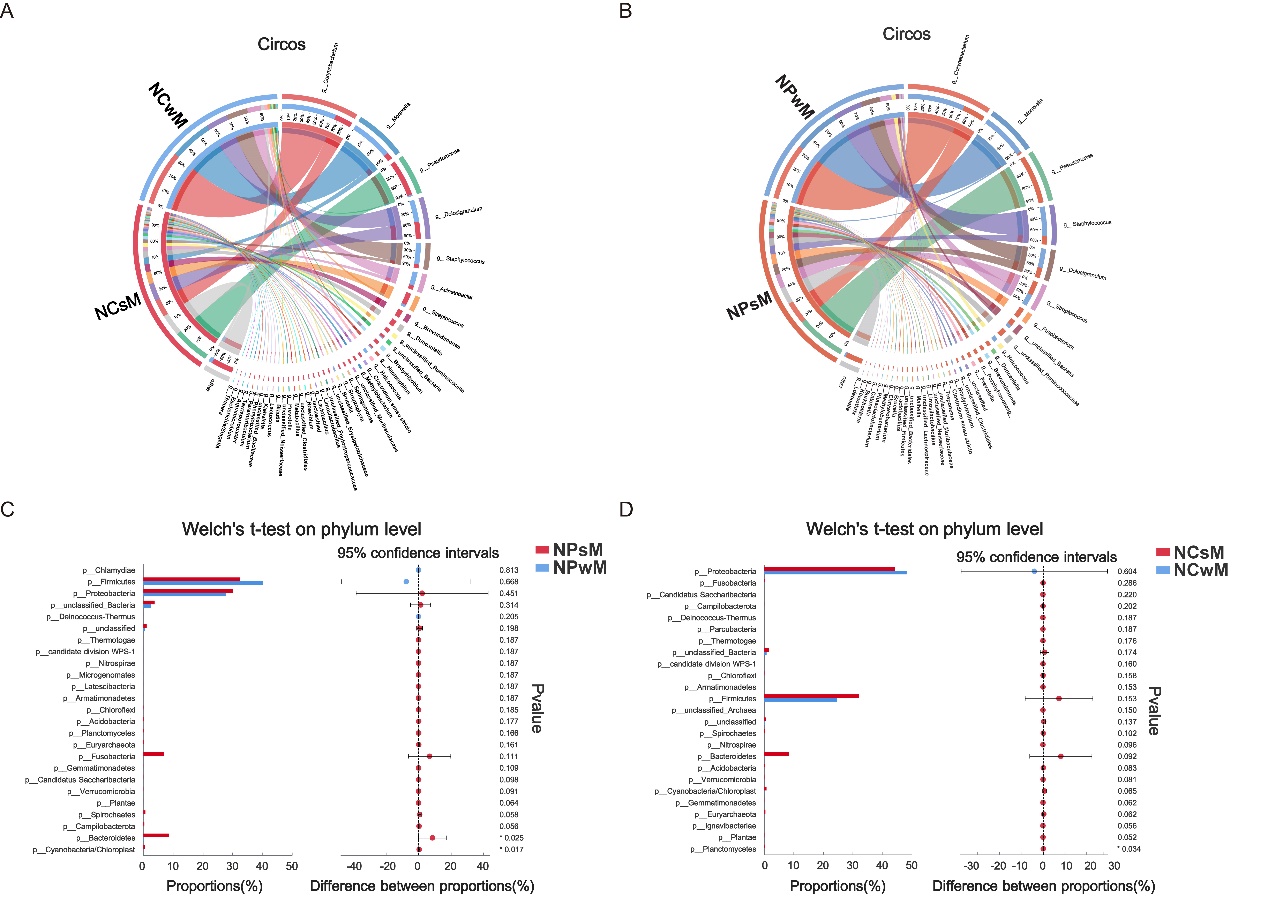


研究结论

在 COVID-19 流行期间，长时间佩戴口罩等隔离措施为我们提供了研究儿童鼻咽和鼻腔微生物群变化的难得机会。健康儿童的微生物群多样性较低，可能伴随着微生物群失调、呼吸道感染风险增加和炎症反应。这项研究强调了重建微生物群平衡的重要性，并突出了在常规公共卫生实践中采取个性化治疗和预防策略的必要性。
